# Immunogenicity and Safety of the Bivalent Respiratory Syncytial Virus Prefusion F Subunit Vaccine in Immunocompromised or Renally Impaired Adults

**DOI:** 10.3390/vaccines13030328

**Published:** 2025-03-19

**Authors:** Natalia Castillo Almeida, Lalitha Parameswaran, Elliot N. DeHaan, Hayley Wyper, Farah Rahman, Qin Jiang, Wen Li, Michael Patton, Maria Maddalena Lino, Zaynah Majid-Mahomed, Elissa Malkin, Matthew Davis, William J. Towner, Kapil Saharia, Kumar Ilangovan, Elena Kalinina, David Cooper, Kena A. Swanson, Annaliesa S. Anderson, Alejandra Gurtman, Iona Munjal

**Affiliations:** 1 Department of Internal Medicine, Division of Infectious Diseases, University of Nebraska Medical Center, Omaha, NE 68198, USA; 2 Department of Internal Medicine, Division of Infectious Diseases, NYU Grossman School of Medicine, New York, NY 10016, USA; 3 Vaccine Research & Development, Pfizer Inc., Pearl River, NY 10965, USAfarah.rahman@pfizer.com (F.R.); kumar.ilangovan@pfizer.com (K.I.);; 4 Vaccine Research & Development, Pfizer Ltd., Marlow SL7 1YL, UK; 5 Vaccine Research & Development, Pfizer Inc., Collegeville, PA 19426, USA; qin.jiang@pfizer.com (Q.J.);; 6 George Washington University Vaccine Research Unit, George Washington University, Washington, DC 20052, USA; 7 Rochester Clinical Research, Inc., Rochester, NY 14609, USA; 8 Southern California Permanente Medical Group, Kaiser Permanente Southern California, Los Angeles, CA 90027, USA; 9 University of Maryland School of Medicine, University of Maryland, Baltimore, MD 21201, USA; ksaharia@ihv.umaryland.edu

**Keywords:** RSVpreF, RSV, immunogenicity, safety, immunocompromised, renally impaired, adults

## Abstract

**Background/Objectives:** Individuals with immunocompromising conditions are at high risk of developing severe respiratory syncytial virus (RSV) illness. This phase 3, single-arm study assessed the safety and immunogenicity of the bivalent RSV prefusion F protein−based (RSVpreF) 120-µg vaccine in immunocompromised and renally impaired adults. **Methods:** Participants were stratified by age group (18−<60-year-olds; ≥60-year-olds) and received two RSVpreF doses 1 month apart (i.e., Dose 1 and Dose 2, respectively). Reactogenicity events were collected for 7 days after each dose; adverse events through 1 month after the last dose; and serious adverse events, adverse events of special interest, and newly diagnosed chronic medical conditions throughout the study. **Results:** One month after Dose 1, RSVpreF elicited robust immune responses overall and across age and immunocompromised subgroups. Overall, geometric mean fold rises from before to 1 month after Dose 1 were high for RSV A and RSV B (8.3 and 9.0, respectively); no additional increases 1 month after Dose 2 (7.5 and 7.8) were observed. The most frequent local reaction was pain at the injection site, which was more common after Dose 2 than after Dose 1. The most frequent systemic event after any dose was fatigue. Most local reactions and systemic events were mild or moderate in severity. Adverse event and serious adverse event rates were 13.5% and 7.3% among 18−<60-year-olds and 22.4% and 14.0% among ≥60-year-olds, respectively. **Conclusions:** A single dose of the RSVpreF vaccine conferred robust immune responses in immunocompromised and renally impaired adults with no safety concerns. (ClinicalTrials.gov Identifier: NCT05842967).

## 1. Introduction

Infection with respiratory syncytial virus (RSV) can lead to a range of clinical presentations, from mild cold-like symptoms to acute respiratory disease with serious, potentially fatal complications, including pneumonia, respiratory failure, and cardiopulmonary exacerbations that may require admission to the intensive care unit (ICU) and mechanical ventilation, particularly among vulnerable populations [1,2].

Immunocompromised individuals are vulnerable to the adverse effects of infectious diseases, such as RSV-associated illness, particularly those individuals who have undergone solid organ transplantation, are receiving immunosuppressive therapy for autoimmune conditions or active treatment for malignancies, or are on hemodialysis because of end-stage renal disease [3,4,5,6,7,8,9,10,11]. These conditions are consistent with those noted by the Centers for Disease Control and Prevention as being at high risk of severe RSV-associated illness and are largely similar to those of influenza and COVID-19 [6,11,12]. Having an immunocompromised status is associated with severe outcomes, including ICU admission and the requirement for invasive ventilation, as well as a high mortality risk from RSV infection, leading to a substantial economic burden on healthcare systems [5,6,10]. Of additional concern is the increasing prevalence of immunosuppression among adults. In the United States, an estimated 6.6% of adults were classified as immunocompromised in 2021, compared with a national estimate of 2.7% in 2013 [13,14]. This increase in US prevalence was suggested to be attributed partially to increasing access to a greater number of efficacious immunosuppressive treatment options, including immunotherapies [13,14].

Collectively, these findings emphasize the substantial importance of the availability of preventative approaches and population-level programs for RSV infection in this vulnerable population of individuals with immunocompromising conditions [5,6]. Unfortunately, although individuals with immunocompromise are highly susceptible to adverse effects from infectious diseases, they are typically poorly represented in vaccine clinical trial populations; therefore, there remains a paucity of evidence to support vaccination recommendations in this vulnerable population [15].

No effective pharmacologic treatments are available for RSV-associated illness, with care focusing on supportive options and prevention [16]. Monoclonal antibody therapy for infants is available for the prevention of RSV-associated illness but is not licensed in adult populations [17,18]. RSV vaccines are available, including the bivalent RSV prefusion F protein−based vaccine (RSVpreF; Abrysvo^TM^; Pfizer Inc, New York, NY, USA), which contains recombinant RSV A and RSV B antigens from the two circulating RSV subgroups [19,20,21]. The design of RSVpreF, including the selection of antigens, has been described previously [22]. RSVpreF received initial regulatory approval for adults 60 years and older on the basis of safety and efficacy data from the pivotal phase 3 RENOIR (RSV Vaccine Efficacy Study in Older Adults Immunized against RSV Disease) trial, in which 89% vaccine efficacy against RSV-associated lower respiratory tract illness with at least three symptoms or signs was shown at the conclusion of the first RSV season [19,21]. RSVpreF was also recently licensed for prevention of RSV illness in adults 18 to 59 years of age who are at increased risk of RSV illness [19]. The US Advisory Committee on Immunization Practices currently recommends all adults 75 years and older and those 60 to 74 years of age who are at increased risk of severe RSV illness receive RSV vaccination [11].

To date, the currently approved RSV vaccines lack published data supporting immune responses and safety and tolerability profiles in the immunocompromised population. For immunocompromised individuals currently not eligible for vaccination, the availability of RSV vaccination in this vulnerable population remains an important unmet need. Therefore, this descriptive phase 3 study evaluated the tolerability, safety, and immunogenicity of RSVpreF in adults at high risk of developing severe RSV disease because of underlying medical conditions or because of immunocompromise. Described here are the results of this study in immunocompromised and renally impaired adults, findings that address the gap in the understanding of immune responses of RSV vaccines in individuals with a broad and diverse range of immunocompromising conditions.

## 2. Materials and Methods

### 2.1. Study Design and Participants

This study in immunocompromised adults was part of the phase 3 RSV MONeT (IMunizatiON Study for AdulTs at Higher Risk of Severe Illness) trial assessing the tolerability, safety, and immunogenicity of the RSVpreF vaccine in adults at high risk of developing severe RSV disease (ClinicalTrials.gov identifier: NCT05842967). This part of the MONeT trial was a single-arm, open-label study in which adults 18 years and older with immunocompromise received two doses (Dose 1 and Dose 2, respectively) of RSVpreF (120 µg [i.e., 60 µg each of RSV A and RSV B antigens]) via intramuscular injection with an interval of 1 month between doses.

Participants immunocompromised due to the following conditions or treatment regimens were enrolled: (1) solid organ transplant recipients (including heart, lung, liver, or kidney) who received their transplant at least 3 months before enrollment and with no acute rejection episodes within the previous 2 months; (2) individuals receiving active immunomodulator therapy for an autoimmune inflammatory disorder at a stable dose (i.e., receiving the same dose for at least 3 months with no changes in the 28 days before enrollment); (3) individuals with advanced non-small cell lung cancer who received initial or maintenance chemotherapy for at least 2 weeks before enrollment, individuals who are treatment-naive and not expected to receive chemotherapy within at least 2 weeks after study vaccinations, or those who received checkpoint inhibitor treatment or targeted drug therapy (i.e., tyrosine kinase inhibitors, including epidermal growth factor receptor [EGFR], anaplastic lymphoma kinase [ALK], c-ros oncogene 1 [ROS1], v-raf murine sarcoma viral oncogene homolog B1 [BRAF], rearranged during transfection [RET], and neurotrophic tyrosine receptor kinase [NTRK] inhibitors) for at least one treatment cycle before enrollment; and (4) individuals on maintenance hemodialysis treatment secondary to end-stage renal disease.

Exclusion criteria included bleeding diathesis or other conditions linked with protracted bleeding, previous severe adverse reaction to vaccination, or history of transplant rejection or posttransplant lymphoproliferative disorder within the past 3 months. Participants could not have received monoclonal antibodies against RSV within the preceding 6 months or any previous RSV vaccination. Participants who received any live and non-live vaccine within 28 days and 14 days before study vaccination, respectively, were excluded, as were those expected to receive any non-study vaccine within 14 days after study vaccination.

The study complied with the protocol and all associated amendments, which were approved by the individual trial sites’ ethics committees or institutional review boards, as well as with ethical principles originating from international guidelines (i.e., Declaration of Helsinki and those of the Council for International Organizations of Medical Sciences), applicable International Council for Harmonisation good clinical practice guidelines, and all relevant laws and regulations, including privacy laws and regulations. All participants were required to provide signed informed consent before their enrollment within the trial.

### 2.2. Objectives, Endpoints, and Assessments

The primary immunogenicity objective was to describe the immune responses elicited by RSVpreF in immunocompromised adults 18 years and older. To assess immunogenicity, all participants had blood drawn before vaccination and at 1 month after Dose 1 and Dose 2. RSV A and RSV B neutralizing antibody titers were measured for each serum sample at each time point. The exploratory assessment of seroresponse was defined as an antibody titer 1 month after Dose 1 or Dose 2 that was at least four times the lower limit of quantitation (LLOQ) for participants who were seronegative at baseline (i.e., baseline titer below the LLOQ) or at least a four-fold rise from baseline to 1 month after Dose 1 or Dose 2 for participants who were seropositive at baseline (i.e., baseline titer above the LLOQ).

The primary safety objective was to describe the safety profile of RSVpreF in adults with immunocompromise. Participants recorded local reactions (which included pain at the injection site, redness, and swelling) and systemic events (which included fever, as well as fatigue, headache, nausea, vomiting, diarrhea, muscle pain, and joint pain) by maximum severity in an electronic diary for 7 days after each dose. Adverse events were collected from informed consent through 1 month after the last dose. Serious adverse events, adverse events of special interest (i.e., diagnosis of acute polyneuropathy, atrial fibrillation, and Guillain-Barré syndrome, as well as preterm delivery or birth and hypertensive conditions of pregnancy), and newly diagnosed chronic medical conditions were collected from informed consent throughout study participation. Adverse events and serious adverse events were categorized using the Medical Dictionary for Regulatory Activities (version 27.0) terminology.

### 2.3. Statistical Analysis

This was a descriptive study without hypothesis testing. The sample size of the study was not based on statistical criteria, with approximately 200 participants expected to be enrolled. Enrollment was monitored to assist in ensuring a distribution of participants across the age ranges (i.e., 18 to <60 years and ≥60 years) and underlying immunocompromising conditions (i.e., solid organ transplantation recipient, receiving immunomodulator therapy for autoimmune inflammatory disorders, receiving per-protocol treatment for advanced non–small-cell lung cancer, and end-stage renal disease on hemodialysis).

Immunogenicity objectives were evaluated in the evaluable immunogenicity population. This population included all participants who received Dose 1 and Dose 2 of the RSVpreF vaccine, had blood collection and at least one valid and determinate assay result within 1 month after Dose 2, and had no major protocol violations. Neutralizing geometric mean titers (GMTs) measured at each time point and the associated two-sided 95% CIs were determined by calculating the group means and CIs on the natural log scale, based on the Student *t* distribution, and then by exponentiating the results. Neutralizing titer geometric mean fold rises (GMFRs) were calculated as the group mean of the difference (later minus earlier time point) of logarithm-transformed assay results and exponentiating the mean, and the associated two-sided 95% CIs were calculated using the Student *t* distribution for the mean difference on the logarithm scale to construct the CIs and then exponentiating the confidence limits. GMTs and GMFRs were also summarized for each age stratum (18 to <60 years and ≥60 years) and by the subgroups of participants with a specific immunocompromising or at-risk condition or according to receipt of mycophenolate mofetil (MMF) among participants who received a solid organ transplant. Seroresponse rates were summarized descriptively with the associated 95% CIs determined using the method of Clopper and Pearson.

Safety and tolerability were assessed in the safety population, which included all enrolled participants who received at least one dose of the RSVpreF vaccine. For each age group, descriptive summary statistics of safety and tolerability endpoints included counts and percentages of participants with the reaction or event along with the associated two-sided 95% CIs determined by the method of Clopper and Pearson.

## 3. Results

### 3.1. Participants

From May 16, 2023, to August 14, 2023, 217 participants were enrolled, and 203 participants (18 to <60 years of age, *n* = 96; ≥60 years of age, *n* = 107) were vaccinated at 11 sites in the United States (Figure S1). Overall, 93.5% (203/217) and 92.6% (201/217) of enrolled participants across both age groups received Dose 1 and Dose 2 of RSVpreF, respectively, and 88.5% (192/217) of participants completed the study.

Overall, 53.7% (109/203) of participants were female, 73.9% (150/203) were White, and 93.1% (189/203) were of non-Hispanic or non-Latino ethnicity (Table 1). The mean (SD) age at receipt of Dose 1 was 58 (12.2) years. The type of immunocompromised condition was generally balanced between age groups. The most common immunocompromised conditions among participants were autoimmune inflammatory disorders on active immunomodulator therapy (47.8% [97/203]) and history of solid organ transplantation (36.9% [75/203]), most commonly previous kidney transplantation (18.7% [38/203]). For participants who had received solid organ transplantation, the median (range) time from transplantation to Dose 1 was 52.9 (3–431) months, and 10.7% (8/75) received Dose 1 within 1 year after their transplantation (Table S1). Participants who had undergone solid organ transplantation received a variety of maintenance immunosuppressive medications, with the most common being calcineurin inhibitors (93.3% [70/75]) and antimetabolite therapy (64.0% [48/75]). The most common immunosuppressive therapies received by participants with autoimmune disorders were disease-modifying antirheumatic drugs (42.3% [41/97]). Two participants with non–small-cell lung cancer had received a tyrosine kinase inhibitor, and three had received immune checkpoint inhibitor treatment.

### 3.2. Immunogenicity

**Figure 1:**
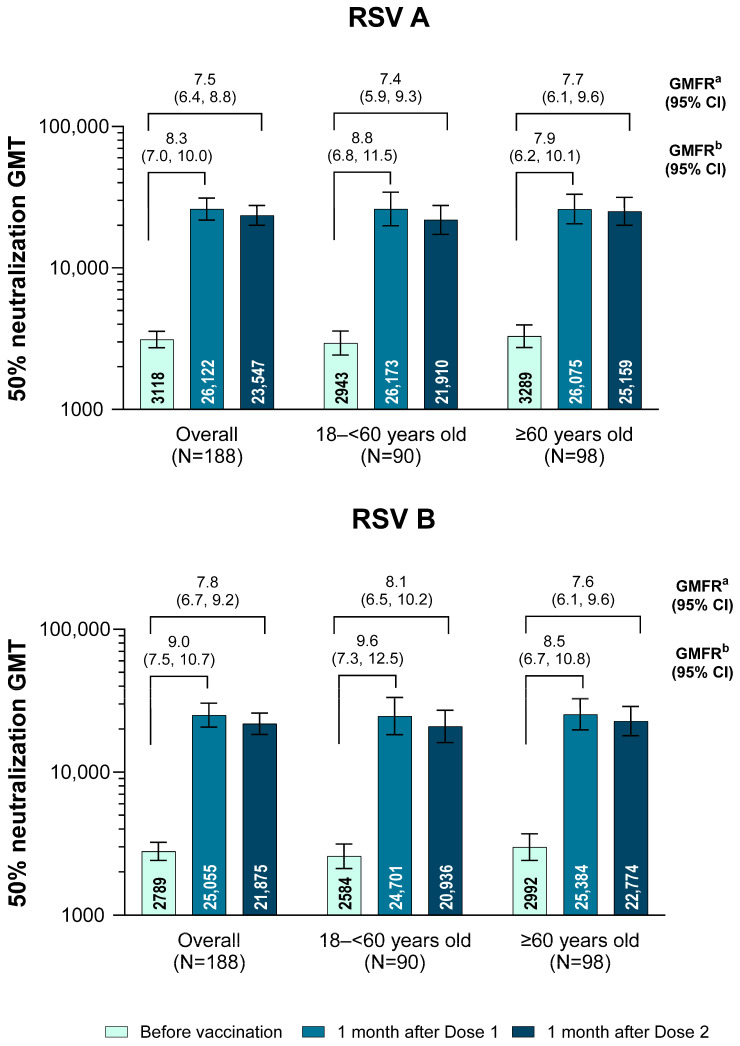
Neutralizing GMTs and GMFRs together with 95% CIs overall and by age group for RSV A and RSV B (evaluable immunogenicity population). The LLOQ values were 242 and 99 for RSV A and RSV B neutralizing titers, respectively. Any assay results that were less than the LLOQ were set to 0.5 × LLOQ for all GMT and GMFR calculations, except for prevaccination assay results less than the LLOQ where the postvaccination result was greater than or equal to the LLOQ, in which case the prevaccination value was then set to the LLOQ when calculating GMFRs. ^a^ GMFR from before to 1 month after Dose 2. ^b^ GMFR from before to 1 month after Dose 1. GMFR = geometric mean fold rise; GMT = geometric mean titer; LLOQ = lower limit of quantitation; RSV = respiratory syncytial virus.

The evaluable immunogenicity analysis population included 188 participants (18 to <60 years of age, *n* = 90; ≥60 years of age, *n* = 98). The RSVpreF vaccine elicited robust immune responses after a single dose, with respective neutralizing GMTs for RSV A and RSV B of 26,122 and 25,055 at 1 month after Dose 1, respectively, and GMFRs from before vaccination to 1 month after Dose 1 of 8.3 and 9.0 (Figure 1). No additional increase was observed 1 month after Dose 2 (neutralizing GMTs of 23,547 and 21,875 for RSV A and RSV B, respectively, 1 month after Dose 2, and corresponding GMFRs from before vaccination to 1 month after Dose 2 of 7.5 and 7.8). Robust immune responses were observed across both age groups (GMFRs from before vaccination to 1 month after Dose 1 for RSV A and RSV B were 8.8 and 9.6, respectively, for 18–<60-year-olds, and 7.9 and 8.5 for ≥60-year-olds). No additional increase was observed after the administration of RSVpreF Dose 2 in either age group. In addition, when excluding those with end-stage renal disease receiving hemodialysis from the overall population who may have less immunocompromise compared with other subgroups, similar immune responses were observed overall and in both age groups (Figure S2).

**Figure 2:**
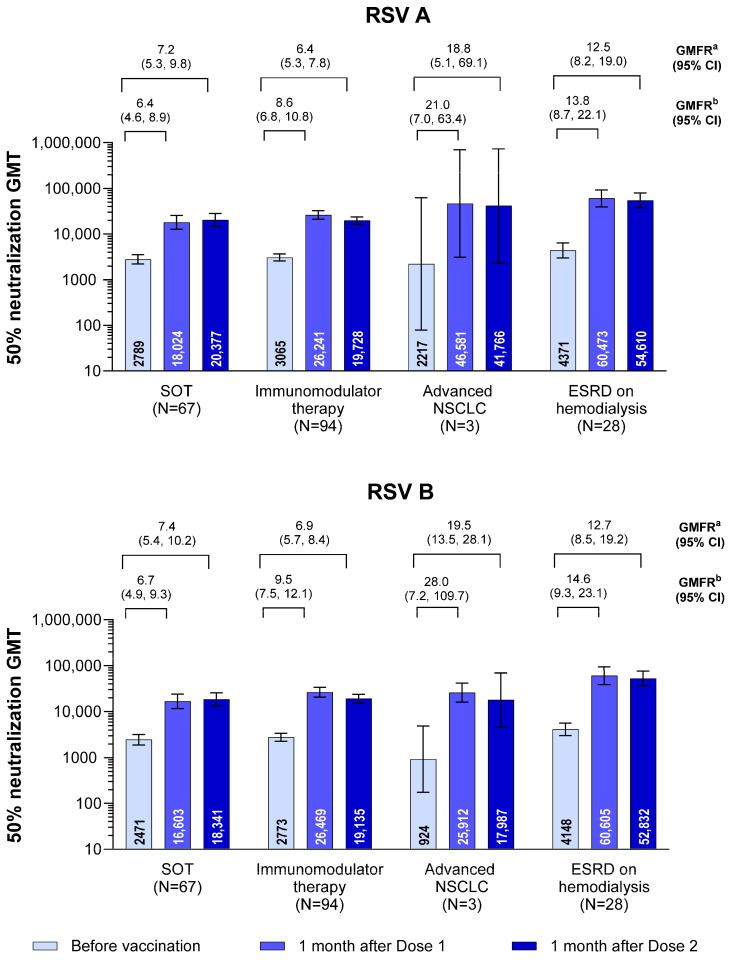
Neutralizing GMTs and GMFRs with 95% CIs by immunocompromising condition for RSV A and RSV B (evaluable immunogenicity population). The LLOQ values were 242 and 99 for RSV A and RSV B neutralizing titers, respectively. Any assay results that were less than the LLOQ were set to 0.5 × LLOQ for all GMT and GMFR calculations, except for prevaccination assay results less than the LLOQ where the postvaccination result was greater than or equal to the LLOQ, in which case the prevaccination value was then set to the LLOQ when calculating GMFRs. ^a^ GMFR from before to 1 month after Dose 2. ^b^ GMFR from before to 1 month after Dose 1. GMFR = geometric mean fold rise; GMT = geometric mean titer; ESRD = end-stage renal disease; LLOQ = lower limit of quantitation; NSCLC = non-small cell lung cancer; RSV = respiratory syncytial virus; SOT = solid organ transplantation.

Neutralizing GMTs prevaccination varied across the subgroups of immunocompromising conditions (Figure 2). However, robust immune responses were observed across immunocompromising conditions after the receipt of RSVpreF. No additional increases in immune responses were observed 1 month after the receipt of Dose 2 of RSVpreF. Among reasonably sized groups, GMFRs from prevaccination to 1 month after Dose 1 were highest in the subgroup of participants who were receiving hemodialysis for end-stage renal disease (*n* = 28; 13.8 for RSV A and 14.6 for RSV B), with lower GMFRs in the subgroup of participants who had received solid organ transplantation (*n* = 67; 6.4 for RSV A and 6.7 for RSV B). For the small number of participants receiving per-protocol treatment for non–small-cell lung cancer (*n* = 3), GMFRs from prevaccination to 1 month after Dose 1 were 21.0 for RSV A and 28.0 for RSV B. GMFRs from prevaccination to after administration of RSVpreF Dose 2 were generally similar compared with after Dose 1 across immunocompromising conditions. In addition, for the subgroup of participants who received solid organ transplantation, those who also received MMF had post-Dose 1 and post-Dose 2 neutralizing titers approximately three times lower than those who did not receive MMF (Figure S3).

**Figure 3:**
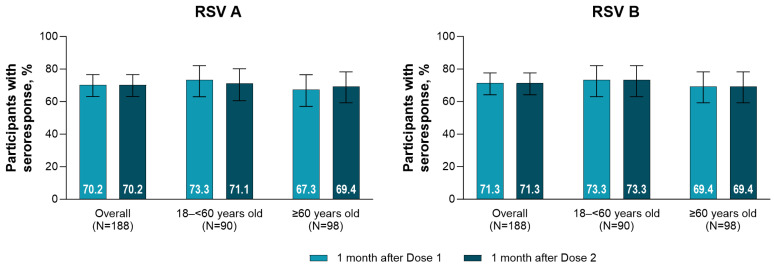
RSV A and RSV B neutralizing titer seroresponse rates 1 month after each RSVpreF vaccination overall and by age group. Data are for the evaluable immunogenicity population. Error bars are the 95% CI. The LLOQ values were 242 and 99 for RSV A and RSV B neutralizing titers, respectively. Seroresponse was defined as achieving a ≥4-fold rise from baseline (before vaccination) if the baseline measurement was above the LLOQ. If the baseline measurement was below the LLOQ, a postvaccination assay result ≥ 4 × LLOQ is considered a seroresponse. LLOQ = lower limit of quantitation; RSV = respiratory syncytial virus; RSVpreF = RSV prefusion F protein−based vaccine.

Seroresponse rates 1 month after RSVpreF vaccination were similar between RSV A and RSV B subgroups, between the younger and older age groups, and between RSVpreF Dose 1 and Dose 2 (Figure 3), as well as when participants with end-stage renal disease receiving hemodialysis were excluded (Figure S4).

Among participants who did not achieve a seroresponse to RSV A (*n* = 56) or RSV B (*n* = 54) after RSVpreF Dose 1, eight participants subsequently achieved an RSV A seroresponse and nine an RSV B seroresponse after Dose 2.

When seroresponse rates were analyzed by subgroup excluding participants with end-stage renal disease receiving hemodialysis, the subgroup of participants who had received solid organ transplantation had lower seroresponse rates than the other subgroups (Figure S5); additionally, seroresponse rates were lower in participants who received solid organ transplantation with MMF treatment versus those not treated with MMF (Figure S6).

### 3.3. Safety

**Figure 4:**
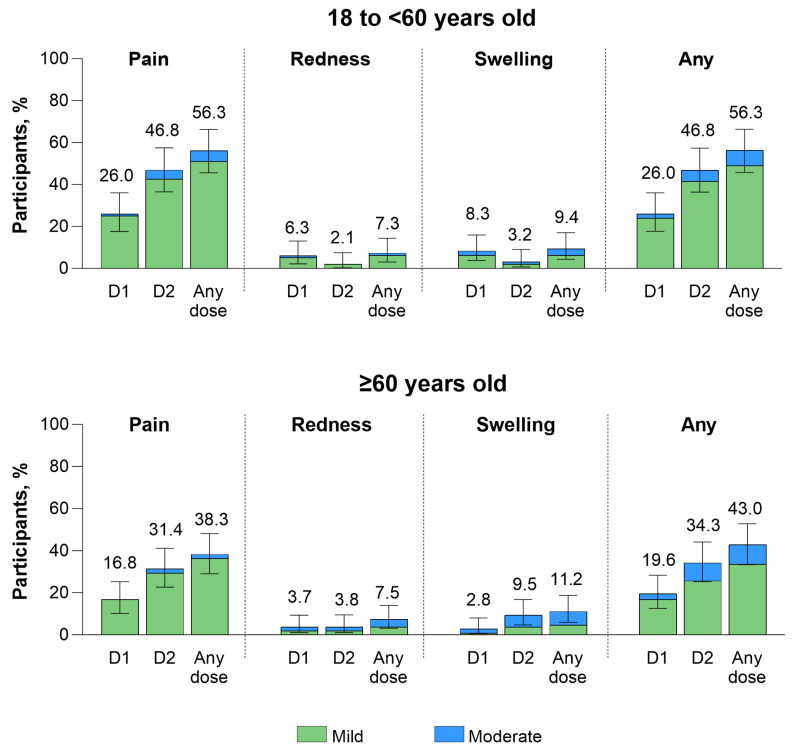
Local reactions occurring within 7 days of RSVpreF vaccination by age group. Data are for the electronic diary safety population, and error bars are the 95% CIs. N values for the 18 to <60 years old group are 96, 94, and 96 for D1, D2, and any dose, respectively. Corresponding values for the ≥60 years old group are 107, 105, and 107. The numbers above the bars are the percentage of participants with the specific local reaction overall. No severe local reactions were observed. The severity rating scale is shown in Table S2. D1 = Dose 1; D2 = Dose 2; RSVpreF = RSV prefusion F protein−based vaccine.

Local reactions after any RSVpreF vaccine dose were more common among the younger (18 to <60 years of age; 56.3%) versus older (≥60 years of age; 43.0%) age group and were more common after Dose 2 than after Dose 1 in both the younger (46.8% after Dose 2 vs. 26.0% after Dose 1) and older (34.3% after Dose 2 vs. 19.6% after Dose 1) age groups (Figure 4). The most common local reaction was pain at the injection site for both the younger (56.3%) and older (38.3%) age groups after any vaccine dose. Pain at the injection site was also more common after Dose 2 than after Dose 1 in both 18 to <60-year-olds (46.8% vs. 26.0%) and ≥60-year-olds (31.4% vs. 16.8%). All local reactions were either mild or moderate in severity. After RSVpreF Dose 1, the onset and duration across all local reactions were 2 to 6 days and 1.5 to 6 days, respectively. The corresponding onset and duration across all local reactions after RSVpreF Dose 2 were 1 to 2 days for both.

**Figure 5:**
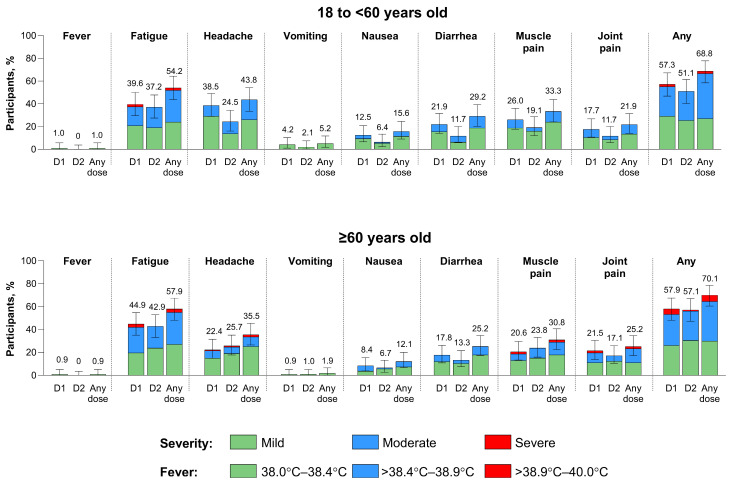
Systemic events occurring within 7 days of RSVpreF vaccination by age group. Data are for the electronic diary safety population, and error bars are the 95% CIs. N values for the 18 to <60 years old group are 96, 94, and 96 for D1, D2, and any dose, respectively. Corresponding values for the ≥60 years old group are 107, 105, and 107. The numbers above the bars are the percentage of participants with the specific local reaction overall. The severity rating scale is shown in Table S2. D1 = Dose 1; D2 = Dose 2; RSVpreF = RSV prefusion F protein−based vaccine.

The frequencies of systemic events after any RSVpreF vaccine dose were similar among the younger (68.8%) and older (70.1%) age groups and similar after Dose 1 and Dose 2 in both the younger (57.3% and 51.1%, respectively) and older (57.9% and 57.1%) age groups (Figure 5). The most common systemic events were fatigue and headache among both the younger (54.2% and 43.8%, respectively) and older (57.9% and 35.5%) age groups. Most systemic events were mild or moderate in severity. Severe systemic events were experienced by 2.1% of participants in the younger age group and 5.6% of participants in the older age group after any RSVpreF vaccine dose. After RSVpreF Dose 1, the onset and duration across all systemic events were 2 to 5.5 days and 1 to 4 days, respectively. The corresponding onset and duration across all systemic events after Dose 2 were 1 to 4.5 days and 1 to 3 days.

**Figure 6:**
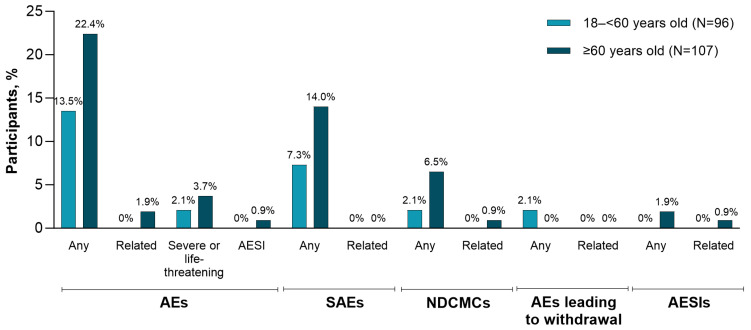
Percentages of participants reporting AEs by age group after receiving the RSVpreF vaccination. Data are for the safety population and are for all AEs excluding prespecified events (i.e., any prespecified events that are electronic diary-specified local reactions and systemic events reported in the AE case report form during the electronic diary collection period, apart from SAEs). AEs were reported through 1 month after the second RSVpreF vaccination; SAEs, NDCMCs, AEs leading to withdrawal, and AESIs are those reported throughout the study. AE = adverse event; AESI = adverse event of special interest; NDCMC = newly diagnosed chronic medical condition; RSVpreF = RSV prefusion F protein−based vaccine; SAE = serious adverse event.

The frequencies of adverse events occurring from RSVpreF Dose 1 to 1 month after Dose 2 were 13.5% among the younger age group and 22.4% among the older age group (Figure 6). Rates of serious adverse events from receipt of RSVpreF Dose 1 through to the end of the study were 7.3% in the younger age group and 14.0% in the older age group; none were considered by the investigator to be related to RSVpreF vaccination. Two adverse events occurring after RSVpreF Dose 1 among participants in the younger age group led to withdrawal from the study (subdural hematoma and kidney transplant rejection). Neither was considered by the investigator to be related to RSVpreF vaccination, and the participant with kidney transplant rejection completed safety follow-up. There was an additional case of antibody-mediated rejection in a participant who had received a lung transplantation; the participant received both RSVpreF doses, the event was not considered by the investigator to be related to study vaccination, and the participant recovered. Throughout the study, one nonserious adverse event of special interest (atrial fibrillation) and one newly diagnosed medical condition (atrial flutter) were reported that were considered by the investigator to be related to RSVpreF vaccination; both events occurred in the same participant in the older age group 16 days after receipt of Dose 1.

## 4. Discussion

The burden of RSV-associated disease in individuals with immunocompromising conditions and renal impairment is substantial, including high rates of mortality and morbidity and healthcare resource use [1,3,4,5,6,7,10,23,24]. Standard-of-care treatment for RSV illness in adults is largely supportive with no licensed preventative monoclonal antibodies or antiviral therapies available [16,18]. In addition, the approved RSV vaccines currently lack published data that support immune responses and safety and tolerability profiles in the immunocompromised population [19,25,26]. The lack of a currently licensed vaccine for the prevention of RSV disease in immunocompromised adults who are too young to be eligible for vaccination is a critical unmet need for this vulnerable population.

In the current descriptive, single-arm, phase 3 study of adults 18 years and older at risk of developing severe RSV disease due to immunocompromising conditions, including receipt of solid organ transplantation, immunomodulator therapy for autoimmune inflammatory disorders, per-protocol treatment for advanced non–small-cell lung cancer, and hemodialysis for end-stage renal disease, a single dose of the RSVpreF vaccine induced robust neutralizing antibody responses. A second RSVpreF dose 1 month after the first dose did not further enhance RSV neutralizing responses across the immunocompromised populations studied. However, a small number of individuals who did not achieve a seroresponse to Dose 1 went on to achieve a seroresponse to Dose 2. In addition, similar robust neutralizing antibody responses were observed regardless of RSV subgroup (i.e., RSV A or RSV B) and in younger (18 to <60 years) and older (≥60 years) age groups. These findings help improve our understanding of the immune responses of RSV vaccines in general and RSVpreF specifically in individuals with a broad and diverse range of immunocompromising conditions.

The robust immune responses we observed after a single RSVpreF vaccination in the current MONeT trial of adults with immunocompromise are consistent with those reported in other phase 3 trials of RSVpreF in adults at high risk of developing severe RSV disease because of older age or related medical conditions [27]. Of note, in an earlier analysis from the phase 3 MONeT trial, a single vaccination with RSVpreF elicited robust neutralizing RSV A and RSV B antibody responses in adults 18 to <60 years of age with conditions putting them at high risk of developing severe RSV disease (e.g., conditions such as chronic pulmonary, cardiovascular, renal, hepatic, neurologic, hematologic, and metabolic disorders), meeting the primary immunobridging noninferiority endpoints from the pivotal phase 3 RENOIR trial in adults 60 years and older in which RSVpreF efficacy against RSV-associated lower respiratory tract illness was demonstrated [27,28]. Consistent with the current analysis from the MONeT trial in adults with immunocompromise, no clinically meaningful differences in RSV neutralizing responses were seen across subgroups of older adults and those with high-risk medical conditions, including by age group and underlying medical condition in either of these earlier phase 3 trials. Two recent US real-world analyses provide additional support for the effectiveness of RSV vaccination in individuals with immunocompromise [29]. Based on a review of electronic health records by the Centers for Disease Control and Prevention and the Veterans Health Administration from the last 4 months of 2023 to the first quarter of 2024, vaccine effectiveness among adults 60 years and older with immunocompromise was 73% (95% CI 48, 85) and 71% (95% CI 52, 83) against RSV-associated hospitalization and RSV-associated emergency department visits/urgent care encounters/acute hospitalizations, respectively.

Immunocompromised individuals can have substantially impaired responses to vaccination, as has been observed for other vaccines, including pneumococcal conjugate and COVID-19 mRNA vaccines [30,31,32,33,34]. For these infectious diseases, additional vaccine doses are required for individuals with immunocompromising conditions [12,35,36]. Therefore, considering the appropriate RSVpreF dosing schedule and need for additional doses for this vulnerable population of adults with immunocompromising conditions was a critical aspect of our study. Our data provide support for the robust immune responses after a single dose of RSVpreF in vulnerable populations, including those who have received solid organ transplantation and those with end-stage renal disease receiving hemodialysis.

In our study, participants who had received solid organ transplantation and those with autoimmune disorders receiving immunomodulator therapy had lower neutralizing GMTs after RSVpreF vaccination than participants with end-stage renal disease receiving hemodialysis. Of note, participants who had received solid organ transplantation together with MMF therapy had lower neutralizing GMTs and seroresponse rates than those who did not receive MMF, after both the first and second RSVpreF doses, though receipt of a second RSVpreF dose did not appreciably affect neutralizing GMTs. We hypothesize that these lower responses may be due to the immunosuppressive medications received, and further study is required to determine the impact of individual therapies in these populations, although the immune responses were still robust and similar to those seen in immunocompetent populations studied previously. Of further note, in some subgroups of participants in our study with specific immunocompromising conditions, high baseline titers suggest high levels of natural exposure, which may have contributed to the differences in immune responses to RSV vaccination as observed in previous clinical trials among healthy adults and those with high-risk conditions [27,37].

The safety and tolerability profiles of RSVpreF after one or two doses in adults with immunocompromising conditions were consistent with those in previous adult studies [28]. Most reactogenicity events were mild or moderate in severity. Local reactions were more common among the younger age group (18 to <60 years of age) than in the older age group (≥60 years of age) and more common after the second rather than the first RSVpreF vaccination. No vaccine-related serious adverse events or vaccine-related adverse events leading to withdrawal were reported. One participant in the ≥60-year age group experienced both an adverse event of special interest (atrial fibrillation) and a newly diagnosed chronic medical condition (atrial flutter) considered by the investigator to be related to RSVpreF vaccination. Two adverse events led to withdrawal, including a kidney transplant rejection; however, neither event was considered related to RSVpreF vaccination. A lung transplant rejection was also reported but was not considered related to vaccination and did not lead to study withdrawal. RSVpreF has also been shown to be safe and tolerable in thousands of adult clinical trial participants without immunocompromising conditions [28,37,38]. Collectively, these data continue to support the favorable safety and tolerability profile of RSVpreF in adult populations. Considering the totality of immunogenicity, safety, and tolerability data for one or two RSVpreF vaccinations as assessed in our study, a single RSVpreF vaccination appears to provide a more favorable benefit-risk ratio in adults with immunocompromising conditions than a two-dose schedule.

Limitations of this analysis include that it was a single-arm, descriptive, phase 3 trial. Therefore, formal immunobridging of the findings to those of the pivotal phase 3 RENOIR efficacy trial is not possible. However, the similar magnitude of immune responses between those observed in the current analysis in adults with immunocompromised conditions and those of adults with high-risk conditions and older adults provides reassurance that RSVpreF will provide consistent protection against RSV-associated illness across these vulnerable adult populations. We also did not investigate different dosing intervals between the first and second RSVpreF vaccine doses and thus cannot determine from this study any potential effect of a longer interval on immune responses and safety and tolerability findings. Another limitation is that this analysis was conducted only in the United States and in adult participants with a limited number of immunocompromising conditions; therefore, the results may not be generalizable to other populations, age groups, or other types of immunocompromising conditions. The effectiveness of RSVpreF should also be elucidated with real-world data following its licensure and implementation within vaccination programs and across a range of immunocompromising conditions. Efficacy of RSVpreF in older adults has been shown through two RSV seasons [21], providing support for the durability of immune responses after a single RSVpreF dose. However, the durability of immune responses has not been investigated in this population of adults with immunocompromise, which will need to be considered in future investigations in terms of the need for revaccination, the interval of which may vary between specific immunocompromised populations (e.g., participants who have received solid organ transplantation with MMF may require a shorter revaccination interval than other immunocompromised populations).

## 5. Conclusions

In conclusion, these results support the use of RSVpreF for the prevention of RSV-associated lower respiratory tract illness in adults with immunocompromising conditions or renal impairment with a single dose, which would fulfill a critical unmet need in this vulnerable population.

## Figures and Tables

**Table 1:** Demographic and clinical characteristics.

Characteristic	RSVpreF 18–<60 Years of Age (N = 96)	RSVpreF ≥60 Years of Age (N = 107)	Total (N = 203)
Age at Dose 1, y			
Mean (SD)	47.8 (9.3)	67.1 (5.0)	58.0 (12.2)
Median (range)	50.5 (23–59)	66 (60–80)	60 (23–80)
Sex, *n* (%)			
Female	56 (58.3)	53 (49.5)	109 (53.7)
Male	40 (41.7)	54 (50.5)	94 (46.3)
Race, *n* (%)			
White	63 (65.6)	87 (81.3)	150 (73.9)
Black	25 (26.0)	15 (14.0)	40 (19.7)
Asian	6 (6.3)	2 (1.9)	8 (3.9)
American Indian, Alaska Native, Native Hawaiian, or other Pacific Islander	2 (2.1)	3 (2.8)	5 (2.5)
Ethnicity, *n* (%)			
Hispanic or Latino	8 (8.3)	3 (2.8)	11 (5.4)
Not Hispanic or Latino	88 (91.7)	101 (94.4)	189 (93.1)
Not reported	0	3 (2.8)	3 (1.5)
Immunocompromised condition, ^a^ *n* (%)	96 (100.0)	107 (100.0)	203 (100.0)
History of SOT ^b^	32 (33.3)	43 (40.2)	75 (36.9)
Heart transplant	1 (1.0)	3 (2.8)	4 (2.0)
Lung transplant	1 (1.0)	20 (18.7)	21 (10.3)
Liver transplant	10 (10.4)	4 (3.7)	14 (6.9)
Kidney transplant	20 (20.8)	18 (16.8)	38 (18.7)
End-stage renal disease on hemodialysis	20 (20.8)	11 (10.3)	31 (15.3)
Autoimmune inflammatory disorders on active immunomodulator therapy ^b^	44 (45.8)	53 (49.5)	97 (47.8)
Oncology history with advanced NSCLC on therapy ^b^	3 (3.1)	2 (1.9)	5 (2.5)
Tobacco use, *n* (%)			
Current	10 (10.4)	7 (6.5)	17 (8.4)
Former	19 (19.8)	57 (53.3)	76 (37.4)
Never	67 (69.8)	43 (40.2)	110 (54.2)
Respiratory rate at baseline, breaths/min			
Mean (SD)	15.9 (2.3)	16.2 (2.6)	16.1 (2.5)
Median (range)	16 (10–23)	16 (10–24)	16 (10–24)

NSCLC = non-small cell lung cancer; RSVpreF = RSV prefusion F protein−based vaccine; SD = standard deviation; SOT = solid organ transplant. ^a^ One participant received concurrent pancreas transplant with kidney transplant, one received dual heart and lung transplant, and one received dual lung and kidney transplant. ^b^ Additional clinical characteristics are summarized in Table S1.

## Data Availability

Upon request, and subject to review, Pfizer will provide the data that support the findings of this study. Subject to certain criteria, conditions, and exceptions, Pfizer may also provide access to the related individual de-identified participant data. See https://www.pfizer.com/science/clinical-trials/trial-data-and-results for more information, accessed on 13 March 2025.

## Supplementary Materials

The following supporting information can be downloaded at: https://www.mdpi.com/article/10.3390/vaccines13030328/s1, Figure S1: Participant flow diagram; Figure S2: Neutralizing GMTs and GMFRs together with 95% CIs overall and by age group for RSV A and RSV B for the population of participants with only immunocompromising conditions; Figure S3: Neutralizing GMTs together with 95% CIs by MMF use in participants who received solid organ transplantation; Figure S4: RSV A and RSV B neutralizing titer seroresponse rates 1 month after each RSVpreF vaccination overall and by age group (excluding participants with ESRD on hemodialysis); Figure S5: RSV A and RSV B neutralizing titer seroresponse rates 1 month after each RSVpreF vaccination by immunocompromising condition (excluding participants with ESRD on hemodialysis); Figure S6: RSV A and RSV B neutralizing titer seroresponse rates 1 month after each RSVpreF vaccination by MMF use in participants who received solid organ transplantation; Table S1: Additional clinical characteristics of participants; Table S2: Severity grading of local reactions and systemic events.
