# Supplementary material for: Immunogenicity and Safety of the Bivalent Respiratory Syncytial Virus Prefusion F Subunit Vaccine in Immunocompromised or Renally Impaired Adults
Source: Vaccines (Basel). 2025 Mar 19;13(3):328. doi: 10.3390/vaccines13030328 (PMC11946143; doi:10.3390/vaccines13030328)
Supplement: Supplementary file 1 [file vaccines-13-00328-s001.zip › Figure S1.pdf]

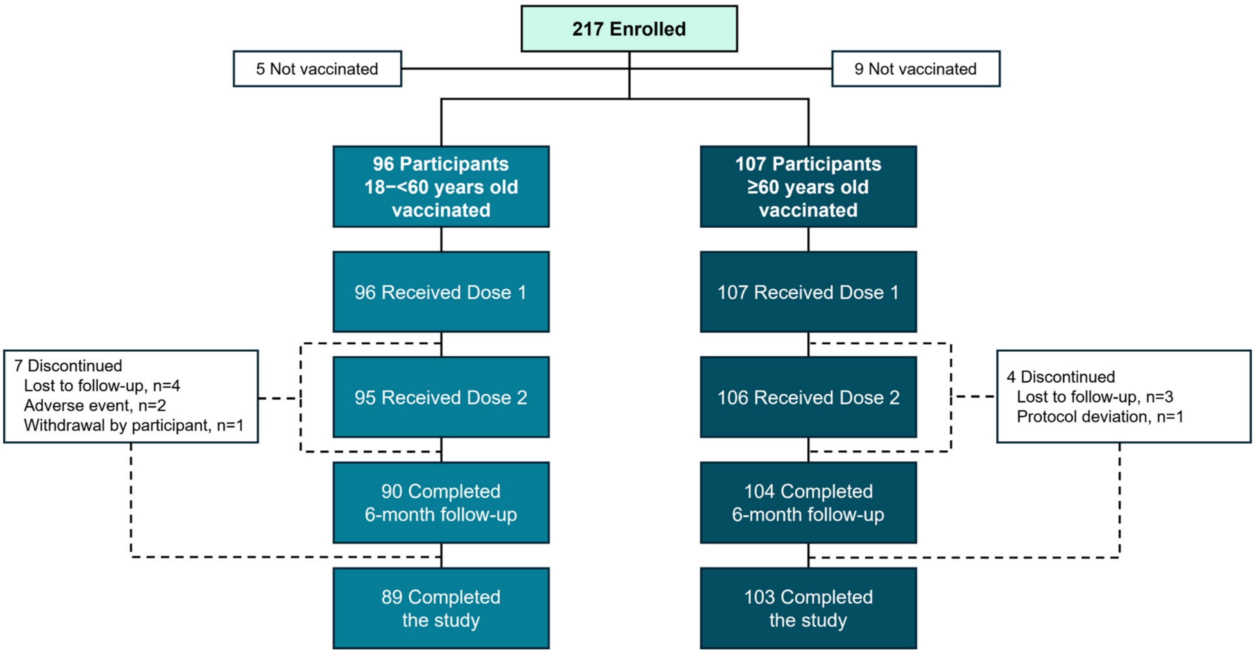

Figure S1. Participant flow diagram.

Participants who completed the study were those who completed both the vaccination and follow-up phases.
