# Supplementary material for: Immunogenicity and Safety of the Bivalent Respiratory Syncytial Virus Prefusion F Subunit Vaccine in Immunocompromised or Renally Impaired Adults
Source: Vaccines (Basel). 2025 Mar 19;13(3):328. doi: 10.3390/vaccines13030328 (PMC11946143; doi:10.3390/vaccines13030328)
Supplement: Supplementary file 1 [file vaccines-13-00328-s001.zip › Figure S2.pdf]

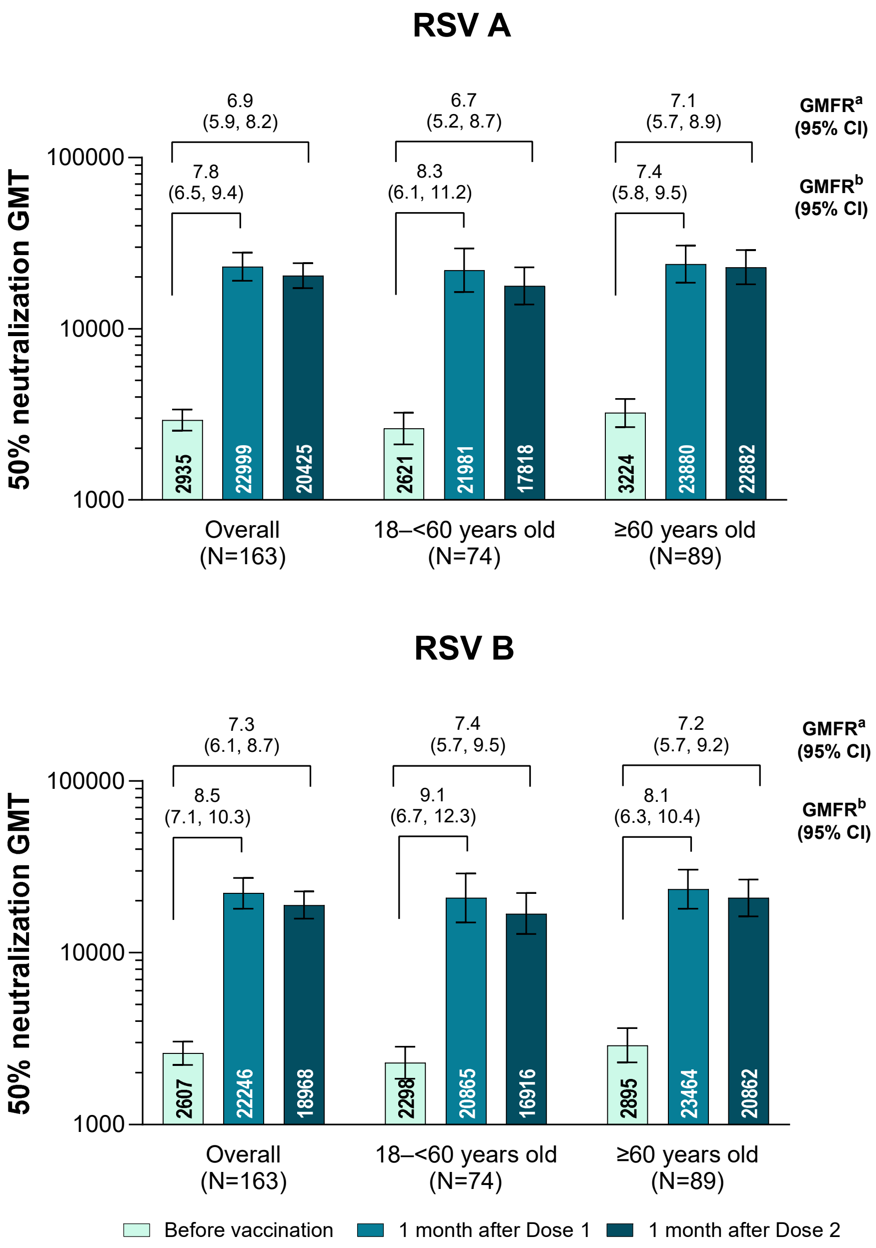

Figure S2. Neutralizing GMTs and GMFRs together with 95% CIs overall and by age group for RSV A and RSV B for the population of participants with only immunocompromising conditions.

Data are for the evaluable immunogenicity population and exclude participants with ESRD receiving hemodialysis. The LLOQ values were 242 and 99 for RSV A and RSV B neutralizing titers, respectively. Any assay results that were less than the LLOQ were set to 0.5 × LLOQ for all GMT and GMFR calculations, except for prevaccination assay results less than the LLOQ where the postvaccination result was greater than or equal to the LLOQ, in which case the prevaccination value was then set to the LLOQ when calculating GMFRs. [a]GMFR from before to 1 month after Dose 2. [b]GMFR from before to 1 month after Dose 1. ESRD = end-stage renal disease; GMFR = geometric mean fold rise; GMT = geometric mean titer; LLOQ = lower limit of quantitation; RSV = respiratory syncytial virus.
