# Supplementary material for: Immunogenicity and Safety of the Bivalent Respiratory Syncytial Virus Prefusion F Subunit Vaccine in Immunocompromised or Renally Impaired Adults
Source: Vaccines (Basel). 2025 Mar 19;13(3):328. doi: 10.3390/vaccines13030328 (PMC11946143; doi:10.3390/vaccines13030328)
Supplement: Supplementary file 1 [file vaccines-13-00328-s001.zip › Figure S3.pdf]

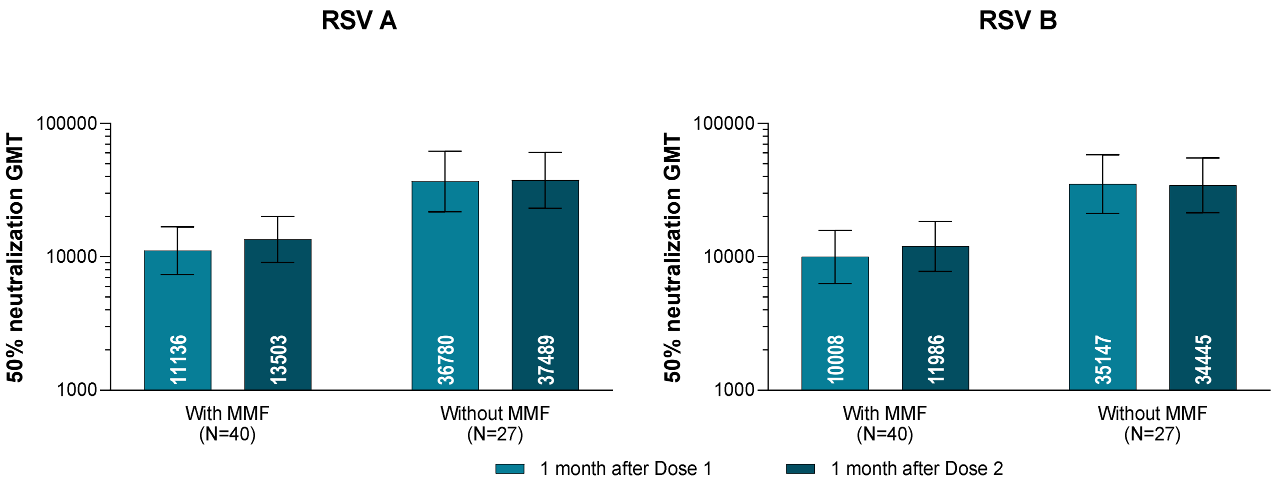

Figure S3. Neutralizing GMTs together with 95% CIs by MMF use in participants who received solid organ transplantation.

Data are for the evaluable immunogenicity population. The LLOQ values were 242 and 99 for RSV A and RSV B neutralizing titers, respectively. Any assay results that were less than the LLOQ were set to 0.5 × LLOQ for all GMT calculations. GMT = geometric mean titer; LLOQ = lower limit of quantitation; MMF = mycophenolate mofetil; RSV = respiratory syncytial virus.
