# Supplementary material for: Immunogenicity and Safety of the Bivalent Respiratory Syncytial Virus Prefusion F Subunit Vaccine in Immunocompromised or Renally Impaired Adults
Source: Vaccines (Basel). 2025 Mar 19;13(3):328. doi: 10.3390/vaccines13030328 (PMC11946143; doi:10.3390/vaccines13030328)
Supplement: Supplementary file 1 [file vaccines-13-00328-s001.zip › Figure S5.pdf]

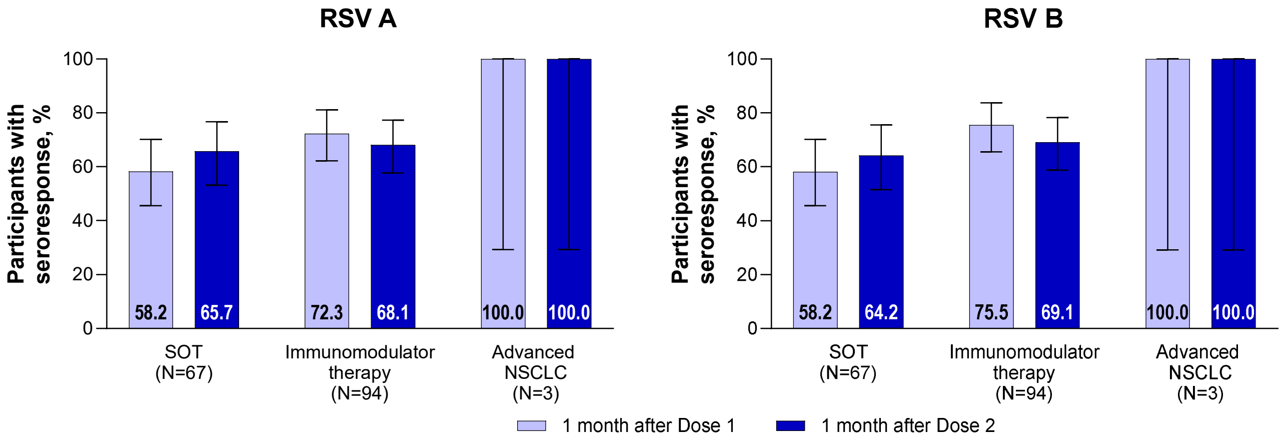

Figure S5. RSV A and RSV B neutralizing titer seroresponse rates 1 month after each RSVpreF vaccination by immunocompromising condition (excluding participants with ESRD on hemodialysis).

Data are for the evaluable immunogenicity population. Error bars are the 95% CI. The LLOQ values were 242 and 99 for RSV A and RSV B neutralizing titers, respectively. Seroresponse was defined as achieving a ≥4-fold rise from baseline (before vaccination) if the baseline measurement was above the LLOQ. If the baseline measurement was below the LLOQ, a postvaccination assay result ≥4 × LLOQ is considered a seroresponse. ESRD = end-stage renal disease; LLOQ = lower limit of quantitation; NSCLC = non-small cell lung cancer; RSV = respiratory syncytial virus; RSVpreF = RSV prefusion F protein–based vaccine; SOT = solid organ transplant.
