# Supplementary material for: Immunogenicity and Safety of the Bivalent Respiratory Syncytial Virus Prefusion F Subunit Vaccine in Immunocompromised or Renally Impaired Adults
Source: Vaccines (Basel). 2025 Mar 19;13(3):328. doi: 10.3390/vaccines13030328 (PMC11946143; doi:10.3390/vaccines13030328)
Supplement: Supplementary file 1 [file vaccines-13-00328-s001.zip › Table S1.pdf]

Table S1. Additional clinical characteristics of participants.

Characteristic	RSVpreF 18–<60 Years of Age	RSVpreF ≥60 Years of Age	Total
**Participants with solid organ transplants**	**N=32**	**N=43**	**N=75**
Time from transplantation to Dose 1			
Mean (SD), months	68.0 (76.2)	84.8 (89.5)	77.6 (83.9)
Median (range), months	54.9 (3–368)	48.1 (9–431)	52.9 (3–431)
<1 year, *n* (%)	6 (18.8)	2 (4.7)	8 (10.7)
1–<5 years, *n* (%)	15 (46.9)	23 (53.5)	38 (50.7)
5–<10 years, *n* (%)	6 (18.8)	8 (18.6)	14 (18.7)
≥10 years, *n* (%)	5 (15.6)	10 (23.3)	15 (20.0)
Maintenance immunosuppressive medication, *n* (%)	32 (100.0)	43 (100.0)	75 (100.0)
Calcineurin inhibitors	29 (90.6)	41 (95.3)	70 (93.3)
Tacrolimus	27 (84.4)	39 (90.7)	66 (88.0)
Cyclosporine	2 (6.3)	3 (7.0)	5 (6.7)
Antimetabolite therapy	22 (68.8)	26 (60.5)	48 (64.0)
Mycophenolate mofetil	21 (65.6)	24 (55.8)	45 (60.0)
Azathioprine	1 (3.1)	2 (4.7)	3 (4.0)
Sirolimus	1 (3.1)	7 (16.3)	8 (10.7)
Everolimus	1 (3.1)	0	1 (1.3)
Belatacept	3 (9.4)	2 (4.7)	5 (6.7)
Prednisone ≥20 mg daily	0	2 (4.7)	2 (2.7)
<1 year from transplantation to Dose 1, *n* (%)	6 (18.8)	2 (4.7)	8 (10.7)
Any induction therapy	6 (18.8)	2 (4.7)	8 (10.7)
Antithymocyte globulin	4 (12.5)	0	4 (5.3)
Basiliximab	4 (12.5)	0	4 (5.3)
Bortezomib	1 (3.1)	0	1 (1.3)
Methylprednisolone	3 (9.4)	2 (4.7)	5 (6.7)
Mycophenolate mofetil	0	1 (2.3)	1 (1.3)

Participants with autoimmune disorders	N=44	N=53	N=97
Active immunomodulator therapy received, *n* (%)			
Azathioprine	2 (4.5)	1 (1.9)	3 (3.1)
Hydroxychloroquine-based regimen	9 (20.5)	9 (17.0)	18 (18.6)
Hydroxychloroquine monotherapy	5 (11.4)	4 (7.5)	9 (9.3)
Hydroxychloroquine + additional agent(s)	4 (9.1)	5 (9.4)	9 (9.3)
Anti-interleukin	14 (31.8)	5 (9.4)	19 (19.6)
Janus kinase inhibitor	0	2 (3.8)	2 (2.1)
Tumor necrosis factor inhibitor	7 (15.9)	15 (28.3)	22 (22.7)
Other concomitant immunosuppressants	7 (15.9)	6 (11.3)	13 (13.4)
Disease-modifying antirheumatic drugs	15 (34.1)	26 (49.1)	41 (42.3)
**Participants with non-small cell lung cancer**	**N=3**	**N=2**	**N=5**
Received cancer therapy, *n* (%)	3 (100.0)	2 (100.0)	5 (100.0)
Alectinib	1 (33.3)	1 (50.0)	2 (40.0)
Nivolumab	2 (66.7)	1 (50.0)	3 (60.0)

RSVpreF = RSV prefusion F protein–based vaccine.
