# Supplementary material for: Immunogenicity and Safety of the Bivalent Respiratory Syncytial Virus Prefusion F Subunit Vaccine in Immunocompromised or Renally Impaired Adults
Source: Vaccines (Basel). 2025 Mar 19;13(3):328. doi: 10.3390/vaccines13030328 (PMC11946143; doi:10.3390/vaccines13030328)
Supplement: Supplementary file 1 [file vaccines-13-00328-s001.zip › Table S2.pdf]

Table S2. Severity grading of local reactions and systemic events.

Event	Mild	Moderate	Severe
Local reactions			
Redness	>2.0–5.0 cm (5–10 measuring device units)	>5.0–10.0 cm (11–20 measuring device units)	>10 cm (>20 measuring device units)
Swelling	>2.0–5.0 cm (5–10 measuring device units)	>5.0–10.0 cm (11–20 measuring device units)	>10 cm (>20 measuring device units)
Injection site pain	Does not interfere with activity	Interferes with activity	Prevents daily activity
Systemic events			
Fatigue	Does not interfere with activity	Some interference with activity	Prevents daily routine activity
Headache	Does not interfere with activity	Some interference with activity	Prevents daily routine activity
Vomiting	1–2 times in 24 hours	>2 times in 24 hours	Requires intravenous hydration
Nausea	Does not interfere with activity	Some interference with activity	Prevents daily routine activity
Diarrhea	2–3 loose stools in 24 hours	4–5 loose stools in 24 hours	≥6 loose stools in 24 hours
Muscle pain	Does not interfere with activity	Some interference with activity	Prevents daily routine activity
Joint pain	Does not interfere with activity	Some interference with activity	Prevents daily routine activity
